# ﻿A new subgenus of *Stigmatophora* Staudinger, 1881 (Lepidoptera, Erebidae, Arctiinae, Lithosiini) from China, with descriptions of three new species

**DOI:** 10.3897/zookeys.1226.138892

**Published:** 2025-02-10

**Authors:** Lu Zhang, Tingting Zhao, Huilin Han

**Affiliations:** 1 School of Forestry, Northeast Forestry University, Harbin 150040, China Northeast Forestry University Harbin China; 2 Northeast Asia Biodiversity Research Center, Northeast Forestry University, Harbin 150040, China Northeast Forestry University Harbin China; 3 Ministry of Education, Key Laboratory of Sustainable Forest Ecosystem Management, Northeast Forestry University, Harbin 150040, China Northeast Forestry University Harbin China

**Keywords:** Checklist, distribution, footman moth, morphology, taxonomy

## Abstract

We describe three newly discovered species of the genus *Stigmatophora* Staudinger, 1881 from China, (*S.bucseki***sp. nov.**, *S.longa***sp. nov.**, and *S.dianensis***sp. nov.**) and establish a new subgenus Bifurcistigma**subgen. nov.** for the first two. Detailed descriptions of the external morphology and male and female genitalia of the new species are provided, along with those of *S.obraztsovi* Daniel, 1951, which is also assigned to the new subgenus. A checklist of all species of *Stigmatophora* is presented including the distribution, type locality, and synonymy of each.

## ﻿Introduction

The genus *Stigmatophora* Staudinger, 1881 belongs to the tribe Lithosiini of the subfamily Arctiinae and was established with *Setinamicans* Bremer & Grey, 1852 as the type species from Beijing (Peking), China (Staudinger 1881). Before 2013, 23 *Stigmatophora* species were known (Bremer and Grey 1852; Walker and Gray 1855; Moore 1878; Meyrick 1894; Swinhoe 1894; Blanford 1898; Leech 1898; Seitz 1911; Wileman 1911; Matsumura 1927, 1930; Daniel 1951; Fang 1991), which are distributed in the northern part of the Oriental region, as well as the central and eastern Palaearctic region (Volynkin et al. 2018; Volynkin 2020). In recent years, *Stigmatophora* has been reviewed and divided into two subgenera based on the characters of male genitalia, *Stigmatophora* and *Pseudomiltochrista* Dubatolov & Bucsek, 2016 (Volynkin et al. 2018; Volynkin 2020; Volynkin et al. 2021), three new species were described. To date, the genus contains 26 species worldwide, and among them, 22 species are found in China.

In this study, we describe two new species (*Stigmatophorabucseki* sp. nov., *Stigmatophoralonga* sp. nov.) with an external morphology closely resembling *S.obraztsovi* Daniel, 1951. The wing pattern and male genitalia of three species do not comply with any known subgenera. Therefore, we propose a third subgenus, Bifurcistigma subgen. nov., to accommodate them. A third new species, *S.dianensis* sp. nov., is placed in the subgenus Pseudomiltochrista Dubatolov & Bucsek, 2016. Additionally, the male genitalia of *S.obraztsovi* are illustrated for the first time, and a checklist of all species in the genus, including their type localities and distributions, is provided.

## ﻿Materials and methods

Specimens of the new species were collected using a 220V/450W mercury lamp. For identification, standard methods for dissection and preparation of the genitalia slides were used (Kononenko and Han 2007). Images of the specimens were taken with a Nikon D700 camera, while the genitalia slides were photographed with an Olympus photo microscope and imaged-stacked using Helicon Focus v. 7.0; figures were further processed in Adobe Photoshop CS6. The terminology of genitalia follows the guidelines set by Volynkin (2024). All type specimens of the new species are deposited in the Insect Collection of Northeast Forestry University (NEFU), Harbin, China.

### ﻿Abbreviations used

**NEFU** Northeast Forestry University, Harbin, China

**TS** type species

**TL** type locality

### ﻿Taxonomic account

**Family Erebidae Leach, 1815**

**Subfamily Arctiinae Leach, 1815**

**Tribe Lithosiini Billberg, 1820**

#### ﻿Genus Stigmatophora

Taxon classificationAnimaliaLepidopteraErebidae

Staudinger, 1881

ECBB18CD-C754-5EF5-971B-6DB8A6E0FD24

Stigmatophora Staudinger, 1881: 399.

##### TS.

*Setinamicans* Bremer & Grey, 1852.

##### TL.

Peking region (China, Beijing).

#### ﻿Subgenus Bifurcistigma subgen. nov.

Taxon classificationAnimaliaLepidopteraErebidae

17E5DF3A-119B-5070-A3A5-01AB7E895D9D

https://zoobank.org/6E96C5DE-EC89-48DC-ACE1-7474AC8B709F

##### TS.

*Stigmatophoraobraztsovi* Daniel, 1951, here designated.

##### TL.

Chekiang, “Berge südlich Wenchow” (China, Zhejiang).

##### Diagnosis.

Unlike members of subgenus Stigmatophora (Figs 7, 8, 19, 25) and *Pseudomiltochrista* (Figs 9–12, 20–22, 26), Bifurcistigma subgen. nov. (Figs 1–6, 13–18, 23–24) has the following characters (details for *Stigmatophora* and *Pseudomiltochrista* are in parentheses, respectively): 1) forewing ground color brown, with an orange band (mostly yellow, with a pattern or spots, partially orange; dark yellow, with rosy veins); 2) ground color of hindwing pale brown to pale blackish brown (mostly dark yellow, partially dark orange; light yellow, with rosy tint); 3) head and patagium orange (mostly yellow, partially red; yellow mixed with red); 4) thorax purple-grey (mostly dark yellow, partially light red and orange; brownish-yellow with red); and 5) abdomen pale brown to pale blackish brown (almost dark yellow, partially light red and orange; light yellow).

Male genitalia: 1) uncus stout (others are thinner, ca 1/2 length of Bifurcistigma subgen. nov.; elongated); 2) valva without harpe (harpe small, thorn-like; broad, crest-like); 3) lamella centralis moderately sclerotized (others with no lamella centralis); 4) sacculus with a medial longitudinal sclerotized band at 1/3 of the length (with no medial process, except in *S.flava*; with a setose thorn-like medial process); 5) juxta with two lateral lobes each side (flat, shield-like; with dorsal process); and 6) cornuti in vesica dense, short, and stout, arranged in a U-shaped band (shortest, almost granular; longest, spine-like, and sparsely arranged).

In the female genitalia: 1) posterior margin of 8^th^ sternite sclerotized and semicircular (membranous; irregularly dentate); 2) ductus bursae extremely broad, strongly sclerotized, roughened with wrinkles (elongate, thin, and weakly sclerotized, and smooth; thin, only sclerotized on left side); and 3) corpus bursae densely covered with spinules, without appendix bursae (other two with a strong spine-like scobination and narrow appendix bursae).

#### ﻿ Stigmatophora (Bifurcistigma) obraztsovi

Taxon classificationAnimaliaLepidopteraErebidae

Daniel, 1951

D4C0596E-2AEC-5B06-89C4-449383C69F0E

**Figures 1–12.**
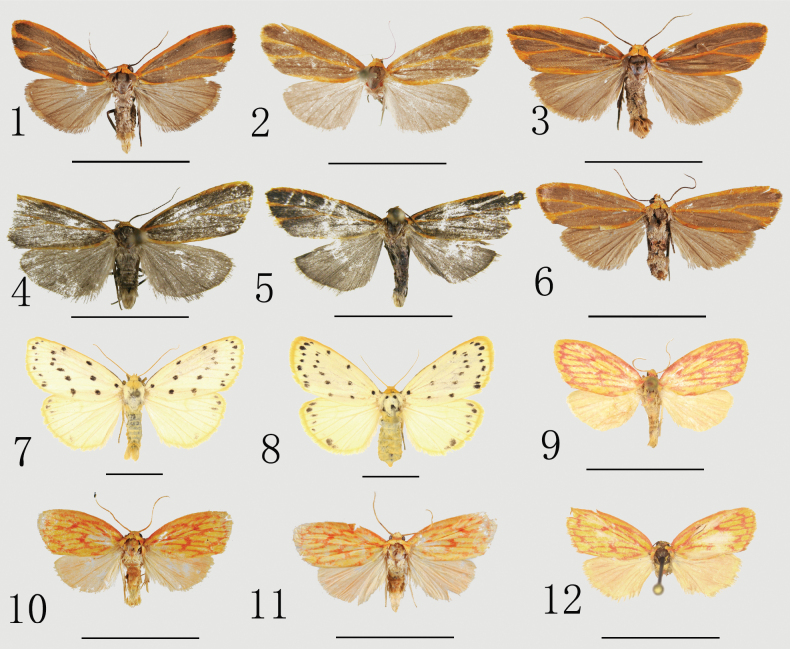
Adults of *Stigmatophora* spp. **1**S. (Bifurcistigma) obraztsovi, male, China (Jiangxi) **2** ditto, female, China (Nanling) **3**S. (B.) bucseki sp. nov., male, holotype, China (Guizhou) **4**S. (B.) longa sp. nov., male, holotype, China (Heilongjiang) **5** ditto, male, paratype, China (Heilongjiang) **6**S. (B.) bucseki sp. nov., female, paratype, China (Guizhou) **7**S. (Stigmatophora) micans, male, Zavkhan aimak, Mongolia (after Volynkin et al. 2018) **8** ditto, female, East Siberia, Russia (after Volynkin et al. 2018) **9**S. (Pseudomiltochrista) zolotuhini, male, India (Assam) (after Volynkin et al. 2018) **10**S. (P.) dianensis sp. nov., male, holotype, China (Yunnan) **11** ditto, male, paratype, China (Yunnan) **12**S. (P.) zolotuhini, female, holotype, Vietnam (Gia Lai) (after Volynkin et al. 2018). Scale bars: 1 cm.

**Figures 13–22.**
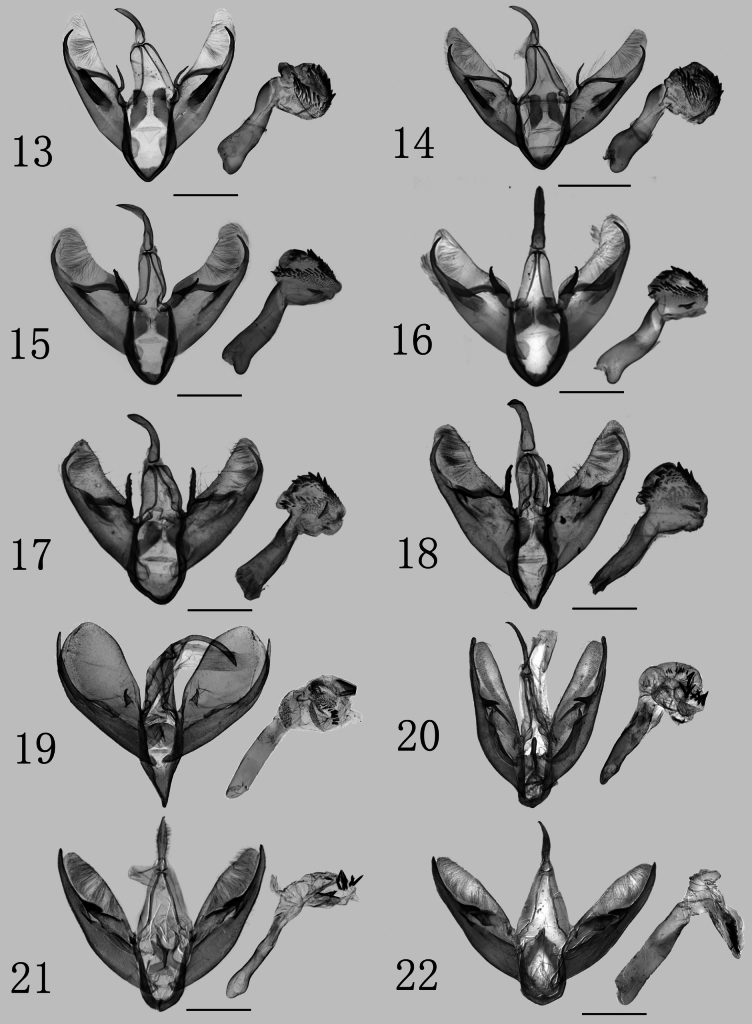
Male genitalia of *Stigmatophora* spp. **13**S. (Bifurcistigma) obraztsovi, genit. prep. no. ztt-436-1 **14** ditto, genit. prep. no. ztt-435-1 **15**S. (B.) bucseki sp. nov., holotype, genit. prep. no. ztt-312-1 **16** ditto, paratype, genit. prep. no. ztt-313-1 **17**S. (B.) longa sp. nov., holotype, genit. prep. no. wp-90-1 **18** ditto, paratype, genit. prep. no. wp-89-1 **19**S. (Stigmatophora) micans (after Volynkin et al. 2018) **20**S. (Pseudomiltochrista) zolotuhini (after Volynkin et al. 2018) **21**S. (P.) dianensis sp. nov., holotype, genit. prep. no. ztt-391-1 **22** ditto, paratype, genit. prep. no. ztt-390-1. Scale bars: 1 mm.

**Figures 23–26.**
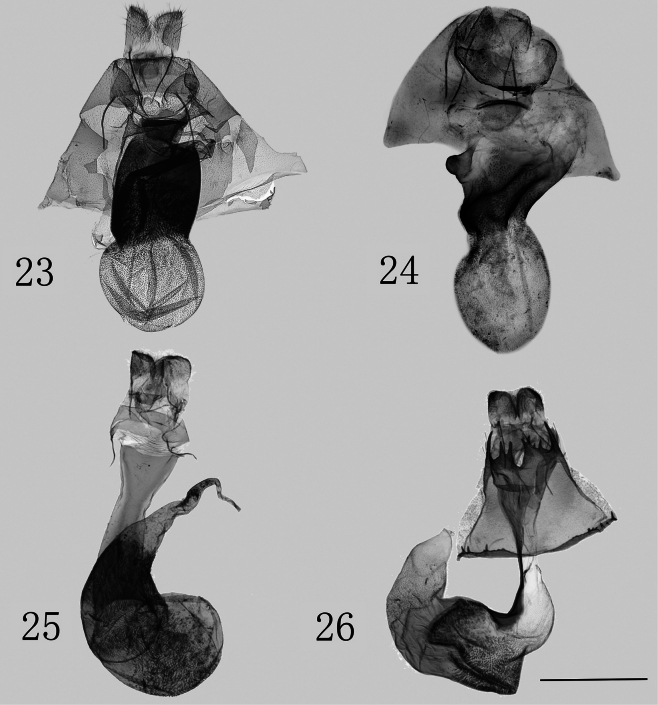
Female genitalia of *Stigmatophora* spp. **23**S. (Bifurcistigma) obraztsovi, genit. prep. no. NSMT-4743 **24**S. (Bifurcistigma) bucseki sp. nov., paratype, genit. prep. no. ztt-397-2 **25**S. (Bifurcistigma) micans (after Volynkin et al. 2018) **26**S. (Pseudomiltochrista) zolotuhini (after Volynkin et al. 2018). Scale bar: 1 mm.

Figs 1 , 2 , 13 , 14 , 23 Common name. 岔带痣苔蛾

Stigmatophora obraztsovi Daniel, 1951: 297, pl. 1, fig. 3.

##### TL.

Chekiang, “Berge südlich Wnchow” (China, Zhejiang).

##### Material examined.

China • 3♂♂; Jiangxi, Jinggangshan, Liujiaping; 3−9.VIII.2021; H. L. Han leg.; genit. prep. no. ztt-435-1, ztt-436-1, ztt-437-1; in NEFU • 1♀; Nanling, 1,000–1,400 m elev., 1–6.VI.2006; M. Wang et al. leg.; genit. prep. no. NSMT-4743, in NSMT.

##### Redescription.

***Adults*** (Figs 1, 2). Forewing length 10 mm in males, 11.6 mm in female. Head and patagium orange, tegula purple-grey; antenna filiform, brownish-yellow, with a dark brown base; thorax purple-grey; abdomen camel-colored, lighter at 1^st^ abdominal segment. Forewing ground color brown, with orange bands along costal margin, inner, and lower margins of cell, and veins M_2_ and Cu_1_; band on M_2_ slightly broadens, and on Cu_1_, it widens toward outer margin, connecting fully with costal margin; orange band on inner margin not reaching anal angle. Hindwing ground color brownish-gray. ***Male genitalia*** (Figs 13, 14). Uncus robust, nearly cylindrical, slightly curved, with a hooked apex. Tegumen weakly sclerotized, thick, ca 1.25 times length of uncus. Valva elongate, narrow, asymmetrical, covered with long hairs, and with a narrowly rounded apex. Costa smooth, slightly raised near base at 1/3 its length. Sacculus weakly sclerotized, broad at base, tapering towards end; ca 7/8 length of valva, middle third rough and band-like, with distal third separated from valva; distal sacculus process curves inward, hook-shaped. Ampulla sclerotized, elongated, with a mound-shaped base. Lamella centralis moderately sclerotized, extending from base of costa to sacculus process. Vinculum strongly sclerotized, wide V-shaped. Juxta with two kidney-shaped lateral lobes on each side, distal half densely covered with small granules, while basal half sclerotized, flat. Aedeagus robust, slightly twisted at middle, tapering towards apex. Carinal region sclerotized. Vesica membranous near base, with a granular field at middle, and a U-shaped band of stout, elongate, triangular cornuti distally. ***Female genitalia*.** (Fig. 23). Papillae anales covered with dense setae. Anterior and posterior apophysis almost equal in length. Ostium bursae strongly sclerotized, rough, and trapezoidal. Ductus bursae cylindrical, extremely broad, and strongly sclerotized, with its interior densely covered with small spines. Corpus bursae membranous, ovoid, rough, and densely covered with small spines that are shorter than those on ductus bursae.

##### Distribution.

China (Fujian, Zhejiang, Jiangxi, Hunan).

#### ﻿ Stigmatophora (Bifurcistigma) bucseki

Taxon classificationAnimaliaLepidopteraErebidae

sp. nov.

7C1B47E7-1C07-5510-AD7C-63BE48921A10

https://zoobank.org/2EA1A963-CD22-49B7-85E5-1BB9E1CCDBFA

Figs 3 , 6 , 15 , 16 , 24 Common name. 布痣苔蛾

##### Material examined.

***Holotype***: China • ♂; Guizhou, Zunyi, Suiyang, Huilong; 6–7.VIII.2020; H. L. Han & J. Wu leg.; genit. prep. no. ztt-312-1; in NEFU.

***Paratypes***: 3♂♂; same data as holotype; H. L. Han & J. Wu leg.; genit. prep. no. ztt-311-1, ztt-313-1, ztt-314-1 • 1♀; Guizhou, Qiandongnan, Leishan, Langde; 18–22.VIII.2020; H. L. Han & J. Wu leg.; genit. prep. no. ztt-397-2; in NEFU • 1♀; Yunnan, Mojiang, Hani Autonomous County; 18−19.IX.2008; H. L. Han & J. Wu leg.; genit. prep. no. ztt-375-2, in NEFU.

##### Diagnosis.

Externally, the new species is similar to S. (B.) obraztsovi (Figs 1, 2, 13, 14, 23), but it can be separated by the following morphological characters (details for S. (B.) obraztsovi are in parentheses): in S. (B.) bucseki, band on vein M_2_ slightly widened toward outer margin and partially connected to costal margin (narrower and fully connected). In the male genitalia, the uncus is long, approximately equal in length to the tegumen (shorter, about 0.75 times); the ampulla is more robust, horn-shaped (slender); and the costa is broader, with the base distinctly enlarged (narrow and smooth). In the female genitalia, the ductus burase is cylindrical (asymmetrical) and the corpus bursae is approximately circular (elliptical).

##### Description.

***Adults*** (Figs 3, 6). Forewing length 9.5 mm in male, 10.7 mm in female. Head and patagium orange; tegula purple-grey; antenna filiform, brown, with dark brown base; thorax purple-grey; abdomen camel-colored, lighter at 1^st^ abdominal segment. Forewing ground color brown, fringe orange; costal, inner, and lower margins of cell, M_2_ and Cu_1_ veins covered with orange band; band on M_2_ slightly widening toward outer margin and slightly connected with costal margin. Hindwing ground color brown, terminal line distinct, fringe camel-colored. ***Male genitalia*** (Figs 15, 16). Uncus robust, moderately sclerotized, nearly sickle, with a spiculate hooked apex. Tegumen weakly sclerotized, thick, same length as uncus. Valva short and wide, with long hairs at apex; costa slightly depressed medially, distinctly widened at base around 1/3 position. Sacculus slightly sclerotized, ca 9/10 length of valva, base wide and tapering towards end, end third separated from valva and hooked; medial third strongly sclerotized, slightly inwardly protruding. Ampulla strongly sclerotized, horn-shaped. Lamella centralis moderately sclerotized, connected with base of ampulla and sacculus process. Vinculum strongly sclerotized, V-shaped. Juxta with two lateral lobes on each side, kidney-shaped, with distal half densely covered small granules. Aedeagus robust and slightly sclerotized. Base of vesica smooth, distal part densely covered in small granules, row of cornuti structures U-shaped; subchamber diverticula with prominent elasma. ***Female genitalia*** (Fig. 24). Papillae anales slightly sclerotized, covered with setae. Anterior and posterior apophysis slender, almost equal in length. 8^th^ sternite sclerotized and semicircular. Ostium bursae slightly sclerotized and rough, scalloped, with a sclerotized, transverse band. Ductus bursae very broad at base and gradually narrowing towards apex, strongly sclerotized, with dense microspines, with conspicuous folds, and a strongly sclerotized, nipple-shaped process on right. Corpus bursae ellipsoidal, rough, and densely covered with small spines.

##### Bionomics.

The type series was collected in early August at an altitude of 958 m in a subtropical mixed forest. The forest floor was densely covered with a variety of shrubs, ferns, and expansive patches of grassland.

##### Etymology.

The new species is named after Slovakian entomologist Karol Bucsek for his help with identification.

##### Distribution.

**Figure 27.**
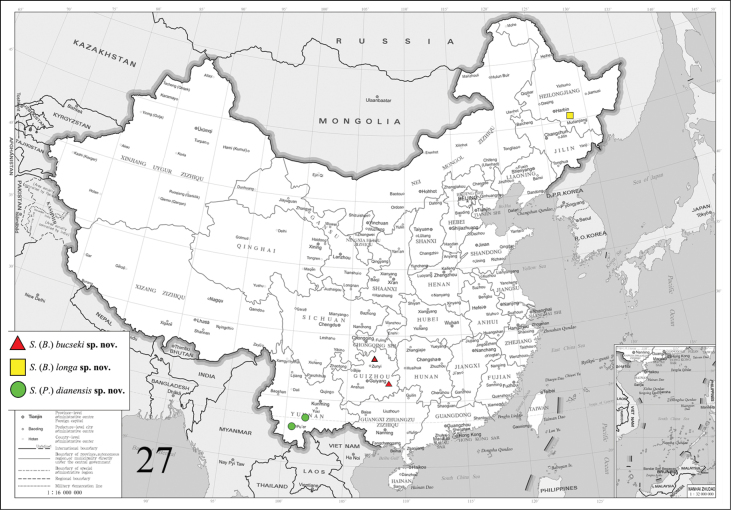
Collecting sites of three new *Stigmatophora* spp.

China (Guizhou: Zunyi, Qiandongnan; Yunnan: Mojiang) (Fig. 27).

#### ﻿ Stigmatophora (Bifurcistigma) longa

Taxon classificationAnimaliaLepidopteraErebidae

sp. nov.

B8E23132-287B-5FE4-8354-BE421D62999F

https://zoobank.org/599DDE26-EF3C-43CF-9098-5DCBADE8C0B4

Figs 4 , 5 , 17 , 18 Common name. 龙痣苔蛾

##### Material examined.

***Holotype***: China • ♂; Heilongjiang, Shangzhi, Changshou, Laoyeling; 5.VIII.2009; P. Wang et.al. leg.; genit. prep. no. wp-90-1, in NEFU.

***Paratype***: 1♂, same data as holotype; genit. prep. no. wp-89-1, in NEFU.

##### Diagnosis.

This new species is highly similar to S. (B.) obraztsovi (Figs 1, 2, 13, 14, 23) and S. (B.) bucseki (Figs 3, 6, 15, 16, 24), but can be distinguished by the following morphological characters (details for S. (B.) obraztsovi and S. (B.) bucseki are in parentheses, respectively): in S. (B.) longa, band on M_2_ not connected with costal margin (completely connected; slightly connected). In the male genitalia, ampulla sclerotized and crutch-shaped (narrow and slender; shorter and horn-shaped).

##### Description.

***Adults*** (Figs 4, 5). Forewing length 9.8 mm in male. Antenna filiform. Thorax dark brown; tegula chocolate; abdomen taupe-brown, lighter on last abdominal segment. Forewing ground color brownish-black, with orange bands along costal, inner, and lower margins of cell and veins M_2_ and Cu_1_; bands along M_2_ and Cu_1_ slightly widening toward outer margin, band along inner margin not reaching anal angle. Hindwing ground color brown, terminal line distinct, fringe brown. ***Male genitalia*** (Figs 17, 18). Uncus sclerotized, robust, and blunt at apex. Tegumen bell-shaped, ca 1.21 times as long as valva. Valva wide, with cucullus rounded. Costa relatively smooth, mid-region slightly concaved. Sacculus sclerotized, wide at base, tapering towards end, sacculus process hooked, separated from valva. Ampulla strongly sclerotized, elongated, crutch-shaped. Lamella centralis moderately sclerotized, extending from base of ampulla to sacculus process. Vinculum strongly sclerotized, V-shaped. Juxta with two weakly sclerotized lateral lobes, kidney-shaped. Aedeagus robust, slightly bent near vesica. Carinal plate weakly sclerotized. Vesica nearly spherical, thin membranous near base, granulated at middle, and with a row of short, distal cornuti distributed. ***Female genitalia*.** Unknown.

##### Bionomics.

The type series was collected in early August at an altitude of 1318 m in a mixed coniferous and broadleaf forest. The forest floor was covered with dense growth of shrubs and trees.

##### Distribution.

China (Heilongjiang: Shangzhi) (Fig. 27).

##### Etymology.

The species is named after “loong”, in honor of the current year of the Chinese zodiac, and it also represents the species collection location, Heilongjiang.

#### ﻿ Subgenus Pseudomiltochrista

Taxon classificationAnimaliaLepidopteraErebidae

Dubatolov & Bucsek, 2016

B4576873-F46D-561D-863D-A6090226BA59

Pseudomiltochrista Dubatolov & Bucsek, 2016: 233.

##### TS.

*Pseudomiltochristazolotuhini* Dubatolov & Bucsek, 2016, by original designation.

##### TL.

Central Vietnam, Gia Lai Prov., K’ Bang Distr., Dak Roong Comm., vill. [age] Kon Loc, Kon Ka Kinh NP [National Park], 14°42.602'N, 108°39.062'E, 1050 m.

#### ﻿ Stigmatophora (Pseudomiltochrista) dianensis

Taxon classificationAnimaliaLepidopteraErebidae

sp. nov.

C276C370-1115-520F-BD6F-B82A43BFF441

https://zoobank.org/1BF307C4-3F94-4FE1-A43D-57FF285D439B

Figs 10 , 11 , 21 , 22 Common name. 云南痣苔蛾

##### Material examined.

***Holotype***: China • ♂; Yunnan, Pu’ er, Simao, Manxieba; 3.VIII.2018; H. L. Han et. al. leg.; genit. prep. no. ztt-391-1; in NEFU.

***Paratypes***: 1♂, same data as holotype; genit. prep. no. ztt-390-1 • 1♂, Yunnan, Xishuangbanna, Jinghong, Mengyang, Wild Elephant Valley; 4−5.VIII.2018; H. L. Han et. al. leg., genit. prep. no. ztt-321-1, in NEFU.

##### Diagnosis.

The new species is similar to S. (P.) zolotuhini (Figs 9, 12, 20, 26), but it can be separated by the following morphological characters (details for S. (P.) zolotuhini are in parentheses): in S. (P.) dianensis, forewing 8 mm long, ovoid, costal and outer margins more arched (10 mm, slightly narrow, smooth). In the male genitalia, uncus short and spindle-shaped (long and slender); apex of apical sacculus process hook-shaped (bluntly finger-shaped); harpe elongate conical, moderately sclerotized, covered with short setae (crest-like, weakly sclerotized); juxta with a flat triangular dorsal process (with a clavate dorsal process); and vesica elongate (fist-shaped, with multiple diverticula).

##### Description.

***Adults*** (Figs 10, 11). Forewing length 8.7 mm in males. Head, patagium, tegula, and thorax dark yellow, with rose veins; antenna filiform, yellow; thorax yellow-brown; abdomen pale yellow. Forewing ovoid, costal and outer margins more arched, with yellow ground color and rose veins, with irregular submarginal line and medial curved spots. Rose-colored pattern completely reaches outer margin. Hindwing pale yellow, scattered with rosy tint. ***Male genitalia*** (Figs 21, 22). Uncus robust, rhombic, with bristle. Tegumen membranous, triangular. Valva narrow and symmetrical; costa membranous and smooth; cucullus rather sharp, covered with rough hairs. Sacculus sclerotized, wide at base, gradually narrowing toward apex, with a strongly sclerotized medial process, sharkfin-shaped, directed downwards. Harpe elongate conical, moderately sclerotized, covered with short setae. Vinculum moderately sclerotized, thick, U-shaped. Juxta flat, with a triangular dorsal process. Aedeagus slender tubular, with swollen caecum. Vesica elongate; central region covered with small graniculi and a cluster of cornuti. ***Female genitalia*.** Unknown.

##### Bionomics.

Wild Elephant Valley in Xishuangbanna is within the Mengyangzi Protection Area of the Xishuangbanna National Nature Reserve. It is north of Jinghong City and flanked by the Lancang River to the west and the Xiaohei River to the east. The terrain comprises low hills and mountain basins. The valley has abundant natural resources, harboring tropical rainforests, South Asian tropical evergreen broadleaf forests, and a diverse array of rare flora and fauna. Specimens were collected in a dense broadleaf forest covered with ferns.

##### Distribution.

China (Yunnan: Pu’ er, Xishuangbanna) (Fig. 27).

##### Etymology.

The species name “dian” is derived from the Chinese character “滇” (Diān), which is the abbreviation for Yunnan Province.

### ﻿Checklist of species in the genus *Stigmatophora* Staudinger, 1881, with synonyms, type locality, and distribution

#### ﻿Subgenus Stigmatophora Staudinger, 1881

The *palmata* species-group

1. S. (S.) cernyi Volynkin, 2020.

TL: Thailand, Mount Doi Phahompok.

Distribution: China (Yunnan); Thailand.

2. S. (S.) chekiangensis Daniel, 1951.

TL: China, Chekiang (Zhejiang: W. Tien-Mu-Shan).

Distribution: China (Gansu, Hunan, Guangxi, Sichuan).

3. S. (S.) danieli Volynkin, Huang & Dubatolov, 2021.

TL: China, Jiangxi: Wuyi Shan.

Distribution: China (Zhejiang, Jiangxi, Hubei, Guangdong).

4. S. (S.) flavogrisea Leech, 1899.

TL: China, Sichuan, N of Kangding.

= *Hypeugoaflavogrisea* Leech, 1899

TL: China, Sichuan, N of Kangding.

Distribution: China (Gansu, Sichuan).

5. S. (S.) hainanensis Fang, 1991.

TL: China, Hainan.

Distribution: China (Guangdong, Hainan, Jiangxi, Guangxi).

6. S. (S.) inanis Seitz, 1913.

TL: Pakistan, Punjab “Murree”.

= *S.palliduspalmata* N. Singh, Kirti & Joshi in Kirti and Singh 2016.

TL: India “Jammu & Kashmir, Patnitop”.

Distribution: Pakistan; India; Nepal.

7. S. (S.) karenkonis Matsumura, 1930.

TL: Formosa, Karenko (China: Taiwan).

= *Miltochristakarenkonis* Matsumura, 1930.

TL: Formosa, Karenko (China: Taiwan).

Distribution: China (Taiwan); Japan.

8. S. (S.) orientalis Daniel, 1951.

TL: China, Chekiang (Zhejiang).

= *Hypeugoaflavogriseaorientalis* Daniel, 1951.

TL: China, Chekiang (Zhejiang: West-Tien-Mu-Shan).

= *Hypeugoaflavogriseaflavogrisea* Daniel, 1951.

TL: China, Chekiang (Zhejiang).

Distribution: China (Shaanxi, Hebei, Jiangsu, Zhejiang, Jiangxi, Sichuan); Japan; Korea.

9. S. (S.) palmata Moore, 1878.

TL: Northern India, Himachal Pradesh.

= *Lyclenepalmata* Moore, 1878.

TL: northern India, Himachal Pradesh “Dharmsala N.W. Himalaya”.

Distribution: China (Yunnan, Xizang); India.

10. S. (S.) ranruna Matsumura, 1927.

TL: China, Taiwan.

Distribution: China (Taiwan).

11. S. (S.) rhodophila Walker, 1864.

TL: China, Shanghai.

= *Barsinerhodophila* Walker, 1865.

TL: China, Shanghai.

Distribution: China (Shanghai).

S. (S.) rhodophila rhodophila Butler, 1879.

TL: China, Shanghai.

= *Miltochristatorrens* Butler, 1879.

TL: Japan, Yokohama.

Distribution: China (Heilongjiang, Jilin, Beijing, Shaanxi, Shanxi, Shandong, Jiangsu, Shanghai, Zhejiang, Henan, Hubei, Hunan, Guangxi, Sichuan); Japan; Korea; Russia.

S. (S.) rhodophila zeyana Dubatolov, 2013.

TL: Russia, Amurskaya Oblast, Zeyskii Nature Reserve (the status is unclear).

Distribution: Russia.

12. S. (S.) roseivena (Hampson, 1894).

TL: Burma, Momeit.

Distribution: China (Jiangxi, Fujian, Hainan); Myanmar; Thailand; Vietnam.

##### Other species with unclear status

13. S. (S.) acerba Leech, 1899.

TL: China, Moupin (Sichuan).

= *Miltochristaacerba* Leech, 1899.

TL: China, Moupin (Sichuan).

Distribution: China (Gansu, Hunan, Guangxi, Sichuan).

14. S. (S.) confusa Daniel, 1951.

TL: China, Yunnan, Li-Kiang.

Distribution: China (Yunnan).

15. S. (S.) conjuncta Fang, 1991.

TL: China, Gansu.

Distribution: China (Gansu, Beijing, Shanxi, Hubei, Guangxi).

16. S. (S.) disticha Meyrick, 1894.

TL: Burma, Shán States, Koni.

= *Ammathodisticha* Meyrick, 1894.

TL: Burma, Shán States, Koni.

= *Asuradisticha* Hampson, 1900.

TL: Burma, Shán States, Koni.

Distribution: Burma; Bhutan.

17. S. (S.) flava Bremer & Grey, 1852.

TL: China, Shanghai.

= *Setinasinensis* Walker, 1854.

TL: China, Shanghai.

= *Setinaochracea* Kindermann, 1855.

TL: China, Altai.

Distribution: China (Heilongjiang, Jilin, Liaoning, Xinjiang, Hebei, Shanxi, Shandong, Jiangsu, Zhejiang, Fujian, Jiangxi, Hubei, Hunan, Guangdong, Guangxi, Sichuan, Guizhou, Yunnan); Kazakhstan; Russia; Japan; North Korea.

18. S. (S.) grisea Hering, 1936.

TL: China, Gansu.

Distribution: China (Gansu).

19. S. (S.) leacrita Swinhoe, 1894.

TL: Japan, Yokohama.

= *Setinaleacrita* Swinhoe, 1894.

TL: Japan, Yokohama.

Distribution: China (Taiwan); Russia; Japan; Korea.

20. S. (S.) likiangensis Daniel, 1951.

TL: China, N. Yunnan, Li-Kiang.

Distribution: China (Yunnan).

21. S. (S.) micans Bremer & Grey, 1852.

TL: China, Peking (Beijing).

= *Setinamicans* Bremer & Grey, 1852.

TL: China, Peking (Beijing).

= *Setinaalbosericea* Moore, 1877.

TL: China, Shanghai.

Distribution: China (Heilongjiang, Jilin, Liaoning, Shaanxi, Inner Mongolia, Beijing, Hebei, Shanxi, Shandong, Jiangsu, Shanghai, Anhui, Henan, Hubei, Sichuan); East Kazakhstan, South Siberia, Amurland, Russia, Mongolia, North Korea.

22. S. (S.) rubivena Fang, 1991.

TL: China, Yunnan.

Distribution: China (Yunnan).

23. S. (S.) strigivenata Hampson, 1894.

TL: Burma, E. Pegu.

= *Eugoastrigivenata* Hampson, 1894.

TL: Burma, E. Pegu.

Distribution: Burma, Bhutan.

24. S. (S.) tridens Wileman, 1910.

TL: China, Kanshirei.

= *Miltochristatridens* Wileman, 1910.

TL: China, Kanshirei.

Distribution: China (Taiwan).

Subgenus Pseudomiltochrista Dubatolov & Bucsek, 2016.

25. S. (P.) dianensis sp. nov.

TL: China, Yunnan.

Distribution: China (Yunnan).

26. S. (P.) zolotuhini Dubatolov & Bucsek, 2016.

TL: central Vietnam.

Distribution: India; Myanmar; Thailand; Laos; Vietnam.

Subgenus Bifurcistigma subgen. nov.

27. S. (B.) bucseki sp. nov.

TL: China, Guizhou.

Distribution: China (Guizhou, Yunnan).

28. S. (B.) obraztsovi Daniel, 1951.

TL: China, Chekiang (Zhejiang).

Distribution: China (Zhejiang, Fujian, Jiangxi, Hunan).

29. S. (B.) longa sp. nov.

TL: China, Heilongjiang.

Distribution: China (Heilongjiang).

## Supplementary Material

XML Treatment for Stigmatophora

XML Treatment for Bifurcistigma

XML Treatment for Stigmatophora (Bifurcistigma) obraztsovi

XML Treatment for Stigmatophora (Bifurcistigma) bucseki

XML Treatment for Stigmatophora (Bifurcistigma) longa

XML Treatment for Subgenus Pseudomiltochrista

XML Treatment for Stigmatophora (Pseudomiltochrista) dianensis

## ﻿Additional information

### Author ORCIDs

Lu Zhang https://orcid.org/0009-0007-5269-3426

Tingting Zhao https://orcid.org/0000-0003-4139-6120

Huilin Han https://orcid.org/0000-0002-2045-6182

### Data availability

All of the data that support the findings of this study are available in the main text.
